# The verbal, non-verbal and structural bases of functional communication abilities in aphasia

**DOI:** 10.1093/braincomms/fcaa118

**Published:** 2020-08-04

**Authors:** Rahel Schumacher, Stefanie Bruehl, Ajay D Halai, Matthew A Lambon Ralph

**Affiliations:** f1 MRC Cognition and Brain Sciences Unit, University of Cambridge, Cambridge CB2 7EF, UK; f2 Department of Neurology, Inselspital, Bern University Hospital, and University of Bern, 3010 Bern, Switzerland; f3 St Mauritius Rehabilitation Centre, 40670 Meerbusch, Germany; f4 Clinical and Cognitive Neurosciences, Department of Neurology, Medical Faculty, RWTH Aachen University, 52074 Aachen, Germany; f5 Division of Neuroscience and Experimental Psychology, University of Manchester, Manchester M13 9PL, UK

**Keywords:** functional communication, non-verbal cognition, aphasia, stroke, voxel-based correlational methodology

## Abstract

The ability to communicate, functionally, after stroke or other types of acquired brain injury is crucial for the person involved and the people around them. Accordingly, assessment of functional communication is increasingly used in large-scale randomized controlled trials as the primary outcome measure. Despite the importance of functional communication abilities to everyday life and their centrality to the measured efficacy of aphasia interventions, there is little knowledge about how commonly used measures of functional communication relate to each other, whether they capture and grade the full range of patients’ remaining communication skills and how these abilities relate to the patients’ verbal and non-verbal impairments as well as the underpinning lesions. Going beyond language-only factors is essential given that non-verbal abilities can play a crucial role in an individual’s ability to communicate effectively. This study, based on a large sample of patients covering the full range and types of post-stroke aphasia, addressed these important, open questions. The investigation combined data from three established measures of functional communication with a thorough assessment of verbal and non-verbal cognition as well as structural neuroimaging. The key findings included: (i) due to floor or ceiling effects, the full range of patients’ functional communication abilities was not captured by a single assessment alone, limiting the utility of adopting individual tests as outcome measures in randomized controlled trials; (ii) phonological abilities were most strongly related to all measures of functional communication and (iii) non-verbal cognition was particularly crucial when language production was relatively impaired and other modes of communication were allowed, when patients rated their own communication abilities, and when carers rated patients’ basic communication abilities. Finally, in addition to lesion load being significantly related to all measures of functional communication, lesion analyses showed partially overlapping clusters in language regions for the functional communication tests. Moreover, mirroring the findings from the regression analyses, additional regions previously associated with non-verbal cognition emerged for the Scenario Test and for the Patient Communication Outcome after Stroke rating scale. In conclusion, our findings elucidated the cognitive and neural bases of functional communication abilities, which may inform future clinical practice regarding assessments and therapy. In particular, it is necessary to use more than one measure to capture the full range and multifaceted nature of patients’ functional communication abilities and a therapeutic focus on non-verbal cognition might have positive effects on this important aspect of activity and participation.

## Teaser

Despite being crucial for activity and participation, the cognitive and neural bases of functional communication abilities in post-stroke aphasia are not well understood. Based on thorough assessments and analyses, Schumacher *et al.* elucidated these key issues and provide guidance for future clinical practice regarding therapy and assessment of these abilities.

## Graphical Abstract

**Graphical Abstract:**
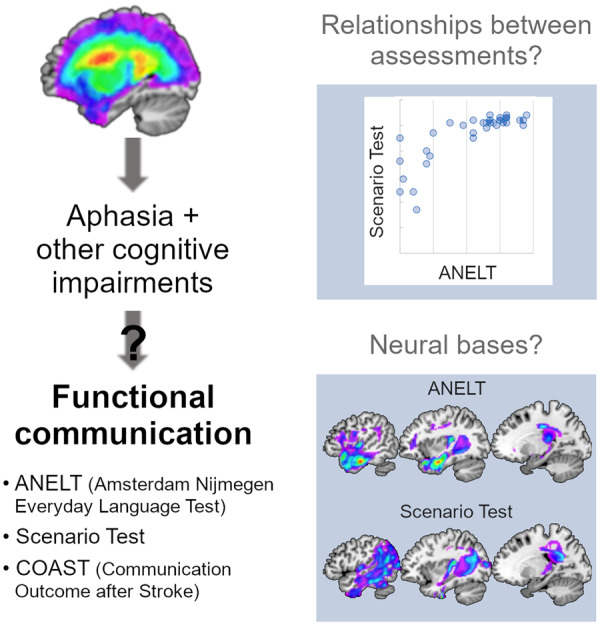

## Introduction

Communication is essential for interactions between individuals. While the most common way to communicate in an everyday setting is via spoken or written language, messages can also be conveyed using other modes, for instance gestures, signs, pictures or assisting devices. Functional communication, defined as ‘the ability to receive and convey messages effectively and independently, regardless of the mode of communication, in natural contexts’ (Fratalli *et al.*, 1995), thus extends beyond language. Therefore, functional communication abilities in individuals with acquired language impairments, as in post-stroke aphasia, not only depend on their language impairment, but also on additional cognitive impairments. It has been shown that additional cognitive impairments are common in individuals with aphasia (Glosser and Goodglass, 1990; Helm-Estabrooks, 2002; Jefferies and Lambon Ralph, 2006; Murray, 2012; Villard and Kiran, 2017; Schumacher *et al.*, 2019) and that they play an important role in recovery (Fillingham *et al.*, 2005; van de Sandt-Koenderman *et al.*, 2008; Lambon Ralph *et al.*, 2010; El Hachioui *et al.*, 2014; Dignam *et al.*, 2017; Geranmayeh *et al.*, 2017; Conroy *et al.*, 2018; Simic *et al.*, 2019). Importantly, outcome after a stroke relates not only to the severity of an impairment *per se* (e.g. language impairment in aphasia), but also to the degree to which this impairment influences activities and participation (e.g. functional communication) (World Health Organization, 2002). The importance of assessing and improving functional communication abilities of patients with aphasia is increasingly recognized (Hilari *et al.*, 2018). Indeed, many recent randomized controlled trials of aphasia include an assessment of functional communication as an outcome measure (Doesborgh *et al.*, 2004; Berthier *et al.*, 2006; de Jong-Hagelstein *et al.*, 2011; Bowen *et al.*, 2012; Sickert *et al.*, 2014; Brady *et al.*, 2016; Breitenstein *et al.*, 2017; Nouwens *et al.*, 2017; Meltzer *et al.*, 2018; Efstratiadou *et al.*, 2019; Palmer *et al.*, 2019). However, despite the importance of communication abilities to everyday life and their primacy in measuring the efficacy of aphasia interventions, there is little knowledge about how commonly used measures of functional communication relate to each other, whether they capture and grade the full range of patients’ remaining communication skills and how these abilities relate to the patients’ cognitive and language impairments as well as to the underpinning lesions.

Several ways to assess functional communication abilities have been developed. Formal tests usually involve some sort of role-play, where a description of an everyday setting is given and participants are required to respond verbally [e.g. the Amsterdam Nijmegen Everyday Language Test (ANELT); Blomert *et al.*, 1994], or both verbally and non-verbally (e.g. the Scenario Test; van der Meulen *et al.*, 2010). The advantage of such tests is that they allow for an objective evaluation of communication in a context that resembles everyday settings. Alternative approaches adopt rating scales, which have the advantage of being based on many instances of the raters’ (patient/carer/therapist) experience but give a more subjective view. The Communication Outcome after Stroke (COAST) rating scale (Long *et al.*, 2008, 2009) is one example of such a rating scale which also includes questions relating to quality of life.

Previous research has used tests or rating scales to elucidate the relationship between functional communication and language impairments (Irwin *et al.*, 2002) and/or other cognitive impairments (Fridriksson *et al.*, 2006; Fucetola *et al.*, 2006; Purdy and Koch, 2006; Murray, 2012; Meier *et al.*, 2017; Olsson *et al.*, 2019; Spitzer *et al.*, 2019), as well as to assess or predict the outcome after stroke (van de Sandt-Koenderman *et al.*, 2008; Blom-Smink *et al.*, 2017). Unsurprisingly, a close association between functional communication and the severity of the language impairment (Fucetola *et al.*, 2006; Meier *et al.*, 2017), especially with phonology or speech production abilities (Irwin *et al.*, 2002; Fridriksson *et al.*, 2006; Blom-Smink *et al.*, 2017), is usually reported. However, the findings are inconclusive with respect to the relationship with performance in non-verbal impairment-level measures. The main reason for the mixed results may lie in the different types of assessments used to measure verbal and non-verbal impairments as well as functional communication.

First, apart from the validation studies (van der Meulen *et al.*, 2010; Hilari *et al.*, 2018), investigations have rarely used more than one measure of functional communication or sampled across the full range and type of aphasias. Thus, some of the inconsistencies in previous findings could stem from differences between functional communication measures and their ability to grade all levels of remaining communication ability. Second, functional communication abilities have been compared with either an overall measure of verbal or non-verbal impairment severity (composite scores of several tests/screening measure; van de Sandt-Koenderman *et al.*, 2008; Meier *et al.*, 2017; Olsson *et al.*, 2019), or to performance in individual tests (Fridriksson *et al.*, 2006; Purdy and Koch, 2006; Murray, 2012). Thus, a more detailed consideration of how functional communication relates to the different components of patients’ verbal and non-verbal cognitive profiles is needed. For instance, a recent study (Schumacher *et al.*, 2019) showed that patients’ performance on a range of standardized tests of attention and executive function was best explained by three orthogonal components (Shift–Update, Inhibit–Generate, Speed), mirroring explorations in healthy participants (Petersen and Posner, 2012; Friedman and Miyake, 2017). These three non-verbal components emerged alongside three orthogonal language components (Phonology, Semantics, Speech Quanta—the amount of speech produced).

In addition to exploring the links between functional communication and combinations of different language and cognitive impairments, it is also unclear how these abilities relate to patients’ lesion profiles. Previous studies have, by means of various lesion–symptom mapping methodologies, examined the brain–behaviour relationships for numerous formal assessments of language and cognitive performance in patients with aphasia (Kummerer *et al.*, 2013; Butler *et al.*, 2014; Mirman *et al.*, 2015; Halai *et al.*, 2017; Lacey *et al.*, 2017; Schumacher *et al.*, 2019). In contrast, none of the studies on functional communication included information on patients’ lesions or formally conducted lesion–symptom mapping with functional communication measures. Beyond providing a better understanding of brain–behaviour relationships, such analyses might yield important information for predicting recovery or for guiding therapeutic interventions.

In this comprehensive investigation, we addressed these key issues by examining functional communication using four different objective and subjective measures across a large patient group, covering a wide range and types of post-stroke aphasia. Due to the availability of detailed verbal, non-verbal and neuroimaging data on the same participants, we were also able to explore the potential link between their variable levels of communication abilities and different aspects of patients’ language ability, attention and executive function, and lesion distributions. Our analyses were designed to address the following currently open questions: (i) How do different measures of functional communication relate to each other across the full range of aphasia severity and types? (ii) Which verbal and non-verbal impairment-level measures relate most to functional communication abilities? (iii) What are the neural bases of functional communication abilities?

## Materials and methods

### Participants

Thirty-seven participants were recruited for the present study (11 female, 26 male; mean age 64 ± 12 years, range: 45–88 years; see Supplementary material). All participants had a single left-hemispheric stroke (ischaemic or haemorrhagic) at least 1 year before assessment and imaging and had no additional significant neurological conditions and no contraindications for MRI. They were pre-morbidly right-handed native English speakers with normal or corrected-to-normal vision. Recruitment took place consecutively from local community clinics and from local NHS referrals. All participants had been diagnosed with aphasia, but no restrictions were applied regarding the type of aphasia or the severity, therefore the sample includes cases ranging from severely to mildly aphasic. Informed consent was obtained from all participants prior to participation, in line with the Declaration of Helsinki and as approved by the local NHS ethics committee.

### Assessments

The data analysed here were collected in two phases, each comprising several sessions within a time frame of around 2 months. Due to the consecutive recruitment of patients, time intervals between testing phases ranged between 5 and 86 months. The first phase included a variety of language production and comprehension tasks as well as some other neuropsychological tests (Butler *et al.*, 2014; Halai *et al.*, 2017). In the second phase, the dataset was enriched with a broad range of standardized neuropsychological tests of attention and executive functions (Schumacher *et al.*, 2019). Performance on all these tests was used to compute the percentage of impaired scores per patient, serving as an indicator of the severity of their verbal and non-verbal impairment, respectively. Moreover, a principal component analysis of these data revealed six orthogonal components, which were previously interpreted as Phonology, Semantics, Speech Quanta, Shift–Update, Inhibit–Generate and Speed (Schumacher *et al.*, 2019). More detailed information, also regarding the time interval between test phases, is provided in the Supplementary material.

The second phase also comprised assessments of functional communication abilities. Two tests and two versions of a rating scale were administered by the first author. The ANELT (Blomert *et al.*, 1994) assesses verbal functional communication and comprises 10 descriptions of everyday situations that are read to the participant, followed by the prompt to say what they would say in that situation. Answers are scored regarding meaningfulness and intelligibility with a maximum total score of 50 each. In this study, only the meaningfulness was analysed. The Scenario Test (van der Meulen *et al.*, 2010; Hilari *et al.*, 2018) similarly uses everyday situations but in contrast to the ANELT, the situations are described and also depicted, and participants are allowed to use any mode of communication they want. Moreover, in case of unsuccessful conveying of the requested information, they are prompted to use a different way of communication. Failing this, closed questions are asked to assess comprehension of the Scenario Test. The test contains six scenarios with three questions each and the maximum score is 54. The COAST is a rating scale assessing perceived communicative effectiveness and quality of life (Long *et al.*, 2008, 2009). The patients’ and the carers’ version each contain 20 questions to be answered on a five-point scale. The last five questions relate to the respondent’s quality of life.

### Statistical analysis

To elucidate the commonalities and differences between the measures of functional communication, correlations between their total scores were computed. For the COAST, sum scores were obtained including as well as excluding the five items on quality of life, as these were the items where patients and carers rated themselves. Analyses that did not include these five items are referred to as Patient/Carer COAST_1–15_. Differences between patients’ and carers’ ratings were analysed by means of paired *t*-tests. The COAST items tap into various aspects of functional communication (Long *et al.*, 2008, 2009). Therefore, sub-scores were derived by applying principal component analyses with Varimax rotation (extraction criterion of Eigenvalue >1) on the Patient and Carer COAST separately. The individual factor scores on each component were then taken as sub-scores and used in further analyses.

The relationship between impairment-level measures and functional communication abilities was elucidated by means of regression analyses. Three approaches including variables of differing specificity were used to relate the (sub)-scores obtained by the tests and rating scales assessing functional communication. The first and broadest approach included the patients’ overall verbal and non-verbal impairment (in percentage of impaired scores in language and non-verbal tests, respectively), similar to previous research using severity measures or very general composite scores. The second, intermediate approach used the more fine-grained factor scores on three verbal components (Phonology, Semantics, Speech Quanta) and three non-verbal components (Shift–Update, Inhibit–Generate, Speed), derived from a principal component analysis (see Schumacher *et al.*, 2019) based on the 32 patients with no missing data. The main advantage of this intermediate approach, in addition to allowing insights beyond the verbal–non-verbal dichotomy, is that the components are orthogonal. Both approaches were carried out in a hierarchical fashion, with lesion volume (being the only patient characteristic significantly correlated with all functional communication measures) entered first, followed by language measures, and lastly by non-verbal measures, to assess the strength of their correlation with functional communication beyond the language impairment itself.

The third and most specific approach sought to identify the most important individual impairment-level measures across the full patient sample. This approach is closest to the reality of a clinical setting where usually only a limited number of measures are available. From the components that were significant in the intermediate-level analysis, we picked tests loading higher than 0.5. These tests were included in a regression analysis with forward selection (criterion of *P* ≤ 0.05 to enter), separately for each functional communication measure. This analysis thus automatically determines which (combination of) variable(s) are most relevant for explaining variance in a variable of interest.

Given the split in patients’ performance on the Scenario Test (see below), we completed an additional exploratory regression analysis (forward selection of intermediate factor scores as independent variables) to elucidate the most important verbal and non-verbal abilities for patients performing either poorly or well on the Scenario Test (splitting the group at a score above or below 50).

### Imaging data acquisition and analysis

High resolution structural T_1_-weighted MRI scans were acquired on a 3.0 Tesla Philips Achieva scanner (Philips Healthcare, Best, the Netherlands) using an 8-element SENSE head coil. A T_1_-weighted inversion recovery sequence with 3D acquisition was employed, with the following parameters: (repetition time) = 9.0 ms, (echo time) = 3.93 ms, flip angle = 8°, 150 contiguous slices, slice thickness = 1 mm, acquired voxel size 1.0 × 1.0× 1.0 mm, matrix size 256 × 256, field of view = 256 mm, (inversion time) = 1150 ms, SENSE acceleration factor 2.5, total scan acquisition time = 575 s.

Structural MRI scans were pre-processed with Statistical Parametric Mapping software (SPM8: Wellcome Trust Centre for Neuroimaging, http://www.fil.ion.ucl.ac.uk/spm/). The images were normalized into standard Montreal Neurological Institute space using a modified unified segmentation–normalization procedure optimized for focal lesioned brains (Seghier *et al*., 2008). Data from all participants with stroke aphasia and all healthy controls were entered into the segmentation–normalization. Images were then smoothed with an 8 mm full width at half maximum Gaussian kernel and used in the lesion analyses described below. An age- and education-matched healthy control group was used to determine the extent of abnormality per voxel. This was achieved using a fuzzy clustering fixed prototypes approach, which measures the similarity between a voxel in the patient data with the mean of the same voxel in the control data (note: this method does not discriminate what caused the abnormality, but simply reflects how deviant the signal in the patient scan is from a healthy group). One can apply a threshold to the fuzzy clustering fixed prototypes to determine membership to abnormal/normal voxel. The default parameters were used apart from the lesion definition ‘U-threshold’, which was set to 0.5 to create a binary lesion image. We modified the U-threshold from 0.3 to 0.5 after comparing the results obtained from a sample of patients to what would be nominated as lesioned tissue by an expert neurologist. The images generated for each patient were visually inspected and manually corrected if necessary and were then used to create the lesion overlap map in Supplementary Fig. 1.

The smoothed fuzzy clustering fixed prototypes negative images (% abnormality) were used to determine the brain regions where abnormality correlated with functional communication measures using a voxel-based correlational methodology (Tyler *et al*., 2005), a variant of voxel-lesion symptom mapping (Bates *et al*., 2003), in which both the behaviour and signal intensity measures are treated as continuous variables (conducted in SPM12). For the structural correlate analysis, we assume a negative correlation between abnormality and behavioural component score (i.e. greater abnormality leads to poorer performance/lower ratings). A separate linear regression model (not including any covariates of no interest) was built for the four functional communication assessments. A voxel-level threshold of *P* < 0.001 and a family-wise error correction at cluster-level *P* < 0.001 were applied, unless noted otherwise. The anatomical labels for the clusters were determined using the Harvard–Oxford atlas for grey matter and on the John Hopkins white matter atlas for white matter tracts.

### Data availability

Behavioural data are available in the Supplementary material. Further data are available by request to the last author.

## Results

Descriptive statistics of all functional communication measures are given in Table 1 and more details are available in the Supplementary material.

**Table 1:** Descriptive statistics of all functional communication measures

	ANELT (*n* = 34)	Scenario Test (*n* = 37)	Patient COAST (*n* = 35)	Carer COAST (*n* = 28)
Mean ± SD	31.4 ± 12.3	45.2 ± 10.5	63.6 ± 13.3	57.1 ± 13.4
Min–max scores (possible range)	10–48 (10–50)	17–54 (0–54)	31.25–87.5 (0–100)	32.5–83.75 (0–100)

ANELT: Amsterdam Nijmegen Everyday Language Test; COAST: Communication Outcome after Stroke; SD: standard deviation.

The separate principal component analyses of the Patient and Carer COAST ratings yielded six components for the patients (accounting for 74.5% of the variance, KMO = 0.61) that were interpreted as: (i) verbal communication, (ii) improvement and participation, (iii) basic communication, (iv) confidence and mood, (v) written language and numbers and (vi) hobbies; and five components for the carers (accounting for 73.9% of the variance, KMO = 0.52), interpreted as: (i) severity, (ii) own quality of life, (iii) written language and numbers, (iv) basic communication and (v) complex interactions. This is generally in line with findings of the validation studies (Long *et al.*, 2008, 2009).

### Relationship between different functional communication assessments

Correlations were computed to elucidate the commonalities and differences between the measures of functional communication. Statistically significant correlations were found among the ANELT, Scenario Test, ratings in the Patient and Carer COAST (with and without items on quality of life), as well as the factor scores from the first component of the Patient COAST (verbal communication), as shown in Table 2.

**Table 2:** Pairwise Pearson correlations between functional communication measures and patient characteristics

	ANELT	Scenario Test	Carer COAST	C COAST_1–15_	C_sub1_: severity	C_sub2_: own quality of life	C_sub3_: written and numbers	C_sub4_: basic communi-cation	C_sub5_: complex inter-actions		Lesion volume	Age	Education	Time post- stroke
**Scenario Test**	0.815**		0.466*	0.442*	0.281	0.272	0.093	0.354	0.048		−0.705**	−0.422**	0.156	−0.051
**ANELT**			0.540**	0.441*	0.381	0.342	0.100	0.021	0.249		−0.741**	−0.329	0.194	−0.016
**Patient COAST**	0.461**	0.228	0.455*	0.422*	0.324	0.154	0.267	−0.123	0.184		−0.208	−0.075	0.058	−0.085
P COAST_1–15_	0.551**	0.323	0.554**	0.529**	0.364	0.165	0.343	−0.054	0.251		−0.348*	−0.193	0.146	−0.087
P_sub1_: verbal communication	0.529**	0.332	0.583**	0.597**	0.563**	0.010	0.224	−0.083	0.327		−0.357*	−0.074	−0.068	−0.019
P_sub2_: improvement and participation	0.175	−0.091	−0.040	−0.144	−0.121	0.294	0.046	−0.280	−0.127		0.150	0.241	−0.010	−0.027
P_sub3_: basic communication	0.309	0.331	0.204	0.049	0.240	0.401*	−0.466*	0.092	0.042		−0.344*	−0.233	0.348*	−0.223
P_sub4_: confidence and mood	−0.134	−0.028	−0.071	0.022	−0.112	−0.226	0.205	0.028	0.044		0.320	0.009	−0.220	−0.027
P_sub5_: written language and numbers	0.065	−0.014	0.262	0.376	0.044	−0.204	0.551*	0.129	0.028		−0.222	−0.243	0.059	0.082
P_sub6_: hobbies	−0.191	−0.278	0.109	0.095	0.183	−0.102	0.048	−0.196	0.196		0.170	0.168	0.110	−0.051
														
**Lesion volume**			−0.626**	−0.603**	−0.589**	−0.159	−0.124	−0.171	−0.113			0.342*	−0.277	0.182
**Age**			−0.105	−0.120	0.032	−0.081	0.011	−0.342	0.049				−0.391*	0.019
**Education**			0.104	0.108	0.080	0.041	−0.072	0.175	−0.187					−0.203
**Time post-stroke**			−0.372	−0.385*	−0.241	0.010	0.126	−0.535**	−0.238					

** P < 0.01.

* *P* < 0.05, two-sided; C/P_sub__(*x*)_ indicates Carer/Patient COAST sub-components (factor scores derived from the principal component analysis).

ANELT: Amsterdam Nijmegen Everyday Language Test; COAST: Communication Outcome after Stroke.

**Figure 1:**
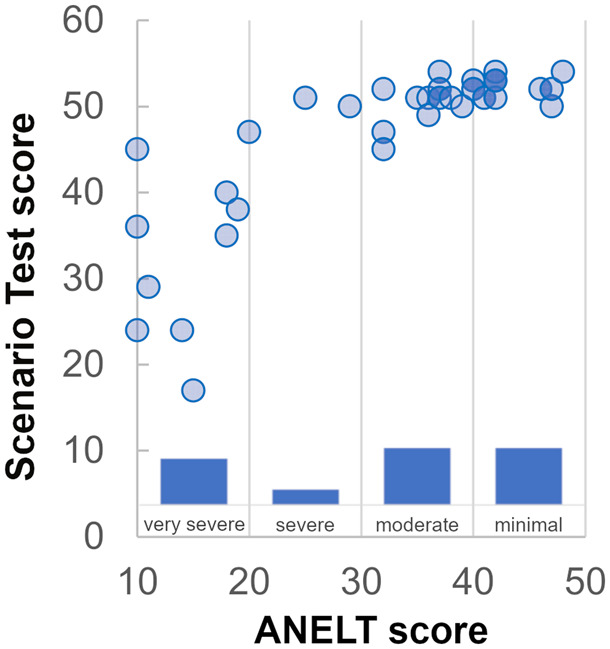
**Relationship between ANELT and Scenario Test scores.** The circles show an individual’s score for both tests. The bars indicate how many individuals fall into which category of severity (based on the ANELT manual).

The strongest correlation was observed between the ANELT and Scenario Test. However, as clearly depicted in Fig. 1, Scenario Test scores were highly variable in patients obtaining low ANELT scores but tended to be at ceiling for patients with higher ANELT scores. The latter, in turn, allowed for a finer grading of patients with high scores in the Scenario Test.

The first component of the Carer COAST (severity) was only correlated with the first component of the Patient COAST (verbal communication) but not with any of the other functional communication scores. Interestingly, there was a significant positive correlation between the second component of the Carer COAST (own quality of life) and patient’s rating of their basic communication abilities. The comparison between the patients’ and carers’ COAST scores (excluding the items on quality of life) revealed a significant difference (*t*(26) = 2.29, *P* = 0.03), with patients rating their communicative abilities higher (mean 61.5 ± 14 standard deviation) than carers (55.7 ± 13.3).

### Relating functional communication abilities to lesion load, verbal and non-verbal status

Regression analyses were performed to elucidate the relationship between impairment-level measures and functional communication abilities. Three approaches, including variables of differing specificity (broad, intermediate, specific), were used to explore their relationship to the functional communication measures. For the broad and intermediate approaches, analyses were carried out in a hierarchical fashion. Lesion volume was entered first, followed by language measures, and lastly by non-verbal measures. Table 3 shows, from left to right, the statistics of all the models computed in this way. For each functional communication (sub)-score (rows), the first three columns of Table 3 show the Adjusted *R*^2^ and the respective *F*- and *P*-values when only lesion load is entered as an independent variable. A *P*-value below 0.05 indicates that the independent variable(s) account for a significant amount of variance in the functional communication measure of interest. The next three columns show the same statistical information but after adding the language variable(s) to the regression model. A further column (sig. *F* change) indicates whether this model is significantly better than the previous one (thus significantly increasing the adjusted *R*^2^). In the last four columns the same is shown for the full models [including the non-verbal variable(s)]. For the specific approach, analyses were carried out using a stepwise forward selection approach and the relevant statistics are given further below in the text.

**Table 3:** Results of the hierarchical regression analyses including lesion volume and broad or intermediate variables of verbal and non-verbal performance

	Adj *R*^2^	*F*(df)	*P*		Adj *R*^2^	*F*(df)	*P*	Sig. *F* change		Adj *R*^2^	*F*(df)	*P*	Sig. *F* change
**Broad**	Lesion volume				+ Verbal impairment					+ Non-verbal impairment			
ANELT	0.535	38.99 (1, 32)	**<0.001**		0.874	115.69 (2, 31)	**<0.001**	**<0.001**		0.876	78.45 (3, 30)	**<0.001**	0.254
Scenario Test	0.483	34.62 (1, 35)	**<0.001**		0.572	25.06 (2, 34)	**<0.001**	**<0.001**		0.606	19.47 (3, 33)	**<0.001**	***0.055***
P COAST_1–15_	0.094	4.54 (1, 33)	**0.041**		0.319	8.96 (2, 32)	**0.001**	**0.002**		0.300	5.85 (3, 31)	**0.003**	0.735
P_sub1_	0.100	4.69 (1, 32)	**0.038**		0.203	5.20 (2, 31)	**0.011**	**0.031**		0.262	4.91 (3, 30)	**0.007**	***0.072***
P_sub2_	−0.008	0.73 (1, 32)	0.399		0.282	7.48 (2, 31)	**0.002**	**0.001**		0.259	4.841 (3, 30)	**0.007**	0.874
C COAST_1–15_	0.336	14.83 (1, 26)	**0.001**		0.314	7.18 (2, 25)	**0.003**	0.798		0.336	5.56 (3, 24)	**0.005**	0.187
C_sub1_	0.320	13.26 (1, 25)	**0.001**		0.296	6.46 (2, 24)	**0.006**	0.724		0.267	4.15 (3, 23)	**0.017**	0.832
C_sub2_	−0.014	0.65 (1, 25)	0.429		0.158	3.43 (2, 24)	**0.049**	**0.021**		0.133	2.33 (3, 23)	0.101	0.573
C_sub4_	−0.010	0.75 (1, 25)	0.394		−0.050	0.39 (2, 24)	0.684	0.829		0.427	7.46 (3, 23)	0.**001**	**<0.001**
					
**Intermediate**	Lesion volume				+ Factor scores verbal components					+ Factor scores non-verbal components			
ANELT	0.508	32.99 (1, 30)	**<0.001**		0.877	56.20 (4, 27)	**<0.001**	**<0.001**		0.968	51.40 (7, 24)	**<0.001**	**0.004**
Scenario Test	0.383	20.27 (1, 30)	**<0.001**		0.607	12.97 (4, 27)	**<0.001**	**0.002**		0.666	9.81 (7, 24)	**<0.001**	***0.078***
P COAST_1–15_	0.068	3.26 (1, 30)	**0.*081***		0.285	4.09 (4, 27)	**0.01**	**0.017**		0.381	3.73 (7, 24)	**0.007**	***0.093***
P_sub1_	0.136	5.73 (1, 29)	0.**023**		0.298	4.18 (4, 26)	**0.010**	**0.039**		0.252	2.45 (7, 23)	**0.050**	0.704
P_sub2_	−0.026	0.25 (1, 29)	0.624		0.202	2.90 (4, 26)	**0.042**	**0.023**		0.321	3.03 (7, 23)	**0.021**	***0.083***
P_sub3_	0.39	2.22 (1, 29)	0.147		−0.066	0.54 (4, 26)	0.711	0.987		0.323	3.05 (7, 23)	**0.020**	**0.004**
C COAST_1–15_	0.247	8.55 (1, 22)	**0.008**		0.157	2.07 (4, 19)	0.125	0.886		0.206	1.85 (7, 16)	0.146	0.282
C_sub1_	0.267	9.02 (1, 21)	**0.007**		0.436	5.26 (4, 18)	**0.006**	***0.053***		0.450	3.57 (7, 15)	0.**018**	0.361
C_sub5_	0.049	2.13 (1, 21)	0.159		0.302	3.38 (4, 18)	**0.031**	**0.036**		0.293	2.30 (7, 15)	***0.083***	0.455

Significant models and *F* changes (*P* < 0.05) are shown in bold, trends (*P* < 0.1) are in italics. For COAST sub-scales, only models which were significant in at least one of the last two steps are shown.

C: Carer; P: Patient; sub: sub-scale.

**Figure 2:**
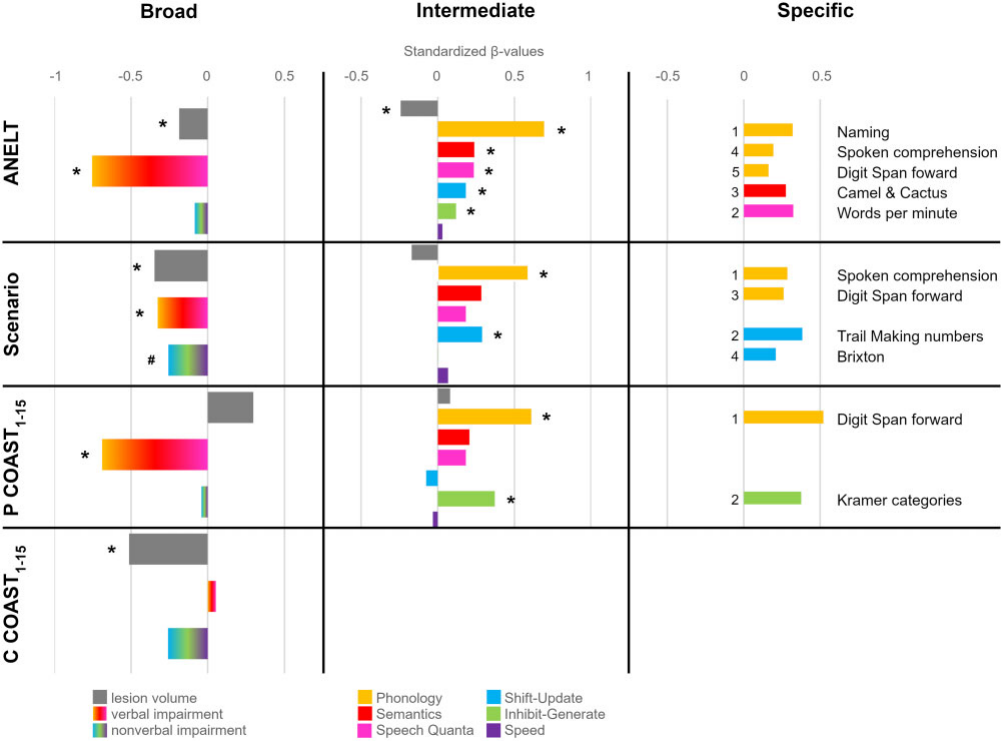
**Standardized *β*-values of the regression models.** Models included one of the functional communication scores as the dependent variable and independent variables on a broad, intermediate and specific level. For simplicity and comparability, the *β*-values of the full models are shown for the broad and intermediate approach if significant, even when the additional variables did not significantly improve the model (see text and Table 3). Lesion volume and verbal/non-verbal impairment have predominantly negative weights because, in contrast to the factor scores in the intermediate approach, a higher score in these measures is considered negative. The numbers in the specific approach indicate the order in which the individual tests were selected by the forward method. P COAST_1–15_/C COAST_1–15_ = Patient/Carer COAST without items on quality of life, *P ≤ 0.05, ^#^*P* = 0.055.

For all three approaches, the contribution of each variable included in the models is shown in Fig. 2, where the standardized versions of the beta weights (or regression coefficients) of all independent variables are depicted [for the broad and intermediate approach, values of the full models are shown but only if they were significant (last *P*-value column in Table 3)]. Standardized beta weights represent the degree to which each independent variable affects the dependent variable if the effects of all the other independent variables are held constant. Thus, a significant beta weight indicates that the variable significantly contributes to the explanation of the variable of interest. The results for the broad approach (including lesion volume and percentage of impairment in verbal and non-verbal tests as independent variables) are shown on the left side of Fig. 2, the results for the intermediate approach [including lesion volume and the factor scores of the three verbal components (Phonology, Semantics, Speech Quanta) and the three non-verbal components (Shift–Update, Inhibit–Generate, Speed)] are shown in the middle, and the results for the specific approach (selecting the most important individual test measures) are shown on the right side of Fig. 2.

Within the broad and intermediate approaches, lesion volume alone was significantly related to all functional communication measures (ANELT, Scenario Test, COAST_1–15_) but the percentage of explained variance varied from around 10% for the Patient COAST_1–15_ to over 50% for the ANELT. Adding the severity of patient’s verbal impairment significantly improved all models apart from the Carer COAST_1–15_. Non-verbal impairment tended to improve the model for the Scenario Test. Similarly, in the intermediate approach, including the verbal independent variables (factor scores of the three verbal components) significantly improved all models apart from the Carer COAST_1–15_, while adding the non-verbal factor scores tended to improve the Scenario Test and Patient COAST_1–15_ regressions and significantly improved the regression with ANELT scores. The importance of non-verbal abilities became further apparent for the COAST sub-scores. Regressions for patients’ and carers’ ratings of basic communication abilities (P_sub3_/C_sub4_—including items such as ‘showing that one does not understand’ or ‘using other ways to communicate’) only became significant once the non-verbal impairment (for carers) or the non-verbal factor scores (for patients) were included in the model. In the latter, the Inhibit–Generate component was the only significantly contributing independent variable. Other COAST sub-scores were more related to language abilities. Phonology was the only significant variable for the verbal communication sub-score of the patient COAST (P_sub1_), while carers based their rating of the severity of the patient’s (verbal) communication difficulties (C_sub1_) on patients’ Speech Quanta. Interestingly, patients’ verbal impairment was in turn the only significant variable for carers’ well-being (C_sub2_).

At the most specific level based on individual test scores, verbal impairment—and Phonology in particular—was the most important variable for the ANELT, Scenario Test and Patient COAST_1–15_, though different tests were most important for each of the functional communication measures. The models for the ANELT, Scenario Test and Patient COAST_1–15_ yielded an adjusted *R*^2^ of 0.92 (*F*(5, 28) = 74.15, *P* < 0.001), 0.78 (*F*(4, 32) = 32.15, *P* < 0.001) and 0.39 (*F*(2, 32) = 11.8, *P* < 0.001), respectively. Interestingly, digit span forward was selected as an important variable for all three measures (see right side of Fig. 2). This simple and short test thus yields important information about verbal communication abilities.

**Figure 3:**
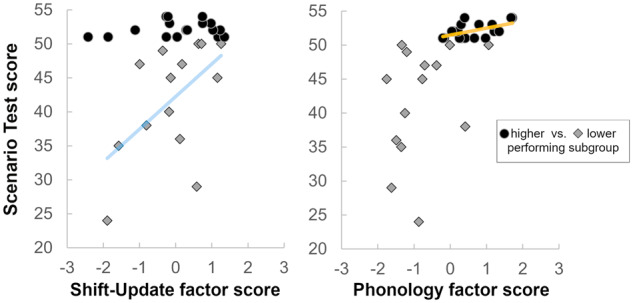
**Relationship between the Scenario Test scores and the two intermediate-level factor scores selected in the subgroup regression analysis.** The sample was split based on the performance in the Scenario Test (lower performing = score of 50 or below, higher performing = score above 50). Only one significant variable per subgroup—Shift–Update for the lower performing subgroup (left) and Phonology for the higher performing subgroup (right)—was selected in the forward selection regression approach, as shown by the respective regression lines.

Given the apparent division within the sample when comparing performance in the ANELT and the Scenario Test, an additional exploratory regression analysis was performed for the two subgroups of patients with high versus lower performance in the Scenario Test. As depicted in Fig. 3, only one intermediate-level independent variable was selected based on forward selection for each group—Shift–Update for the lower performing group *R*^2^ of 0.304 (*F*(1, 12) = 5.25, *P* = 0.04), and Phonology for the higher performing group *R*^2^ 0.299 (*F*(1, 16) = 6.83, *P* = 0.02).

### Brain–behaviour mapping of functional communication abilities

**Figure 4:**
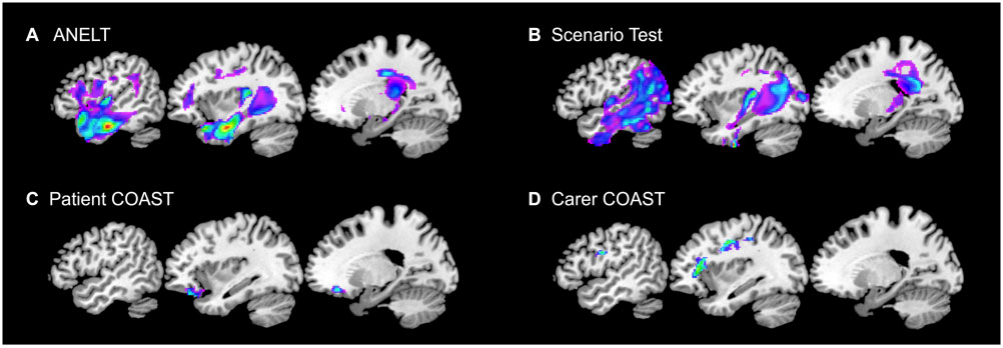
**Structural correlates associated with the functional communication measures.** Separate voxel-based correlational methodology analyses were carried out and the significant clusters for the ANELT (**A**), Scenario Test (**B**), Patient COAST (**C**) and Carer COAST (**D**) are shown. All clusters were obtained by applying a voxel-level threshold of *P* ≤ 0.001 and a family-wise error correction of *P* ≤ 0.001. Montreal Neurological Institute coordinates from left to right are *x* = −50, −36, −20. Figures are in neurological convention (left is left) and thresholded at the respective minimum/maximum *t*-values.

To elucidate whether there are associations between a patient’s lesion and functional communication abilities, we performed separate voxel-based correlational methodology analyses for each functional communication measure. Significant clusters emerged for all measures, as depicted in Fig. 4 and detailed in Table 4. The cluster where tissue abnormality was associated with performance in the ANELT was mainly in the temporal lobe (including the temporal pole) but comprised also frontal (including inferior frontal gyrus) and parietal structures. The Scenario Test cluster overlapped with the ANELT cluster in the temporal lobe but extended more posteriorly into the lateral occipito-temporal cortex and into the parietal cortex. In addition, the Scenario Test cluster included right hemisphere structures, and both clusters contained subcortical regions (mainly parts of the left thalamus). In line with the results from the regression analyses, the ANELT cluster covers more classical language regions while a posterior part of the Scenario Test cluster has also been associated with performance in the non-verbal Shift–Update component (Schumacher *et al.*, 2019). and is generally thought to play an important role in demanding visuo-spatial processing (Fedorenko *et al.*, 2013; Humphreys and Lambon Ralph, 2017). The Patient COAST was associated with a cluster in the (orbito)frontal cortex, while the Carer COAST cluster included more dorsal frontal and parietal structures.

**Table 4:** Clusters and peaks associated with the functional communication measures

Measure	Extent	Location	L/R	*Z*	*x*	*y*	*z*
**Scenario Test**	17 437	Temporal fusiform cortex ant	L	5.84	−36	−8	−44
		Temporal fusiform cortex pos	L	5.34	−38	−34	14
		Temporal fusiform cortex pos	L	5.32	−44	−44	−18
		Planum temporale	L	5.21	−36	−30	14
		Lateral occipital cortex sup	L	5.18	−42	−64	28
		Inferior temporal gyrus post	L	5.15	−46	−32	−16
		Angular gyrus	L	5.07	−42	−60	20
		Precuneus cortex	L	4.97	−26	−58	24
		Angular gyrus	L	4.71	−48	−60	18
		Middle temporal gyrus temocc	L	4.67	−50	−58	−4
		Angular gyrus	L	4.63	−46	−54	26
		Insular	L	4.60	−34	−24	4
	1807	Superior temporal gyrus ant	R	4.71	60	−4	−10
		Parietal operculum cortex	R	4.71	62	−26	18
		Angular gyrus	R	4.64	56	−50	32
		Angular gyrus	R	4.60	56	−46	28
		Supramarginal gyrus ant	R	4.55	68	−22	18
		Planum polare	R	4.48	56	4	−6
		Planum temporale	R	4.46	64	−10	2
		Heschls gyrus	R	4.40	54	−18	8
		Precentral gyrus	R	4.33	60	0	14
	1314	Occipital fusiform gyrus	R	4.60	36	−64	−18
		Brainstem		4.57	12	−42	−18
		Brainstem		4.27	10	−36	−24
		Lateral occipital cortex inf	R	3.79	34	−88	−30
		Lateral occipital cortex inf	R	3.78	40	−84	−16
		Fusiform cortex temocc	R	3.44	46	−46	−24
		Lateral occipital cortex inf	R	3.40	38	−86	−26
	1021	Cingulate gyrus pos	R	4.44	16	−46	26
		Cingulate gyrus pos	R	3.88	8	−26	30
		Cingulum cingulate	R	3.84	12	−34	30
		Lateral ventricle	R	3.79	24	−42	18
		Lateral ventricle	R	3.77	24	−38	20
		Inferior frontal occipital fas	R	3.70	30	−48	16
**ANELT**	13 677	Inferior temporal gyrus post	L	5.57	−48	−14	−24
		Inferior longitudinal fas	L	5.36	−34	−4	−26
		Inferior temporal gyrus post	L	5.18	−44	−26	−18
		Temporal pole	L	5.12	−38	20	−24
		Temporal pole	L	4.92	−46	18	−20
		Inferior longitudinal fas	L	4.89	−44	−30	−16
		Heschls gyrus	L	4.79	−34	−28	14
		Temporal fusiform cortex pos	L	4.79	−34	−10	−34
		Planum temporale	L	4.56	−38	−34	14
		Temporal fusiform cortex ant	L	4.56	−34	−8	−40
		Pallidum	L	4.55	−12	−6	−4
		Inferior longitudinal fas	L	4.52	−42	−2	−32
**Patient COAST_1–15_**	423	Frontal orbital cortex	L	4.13	−22	34	−14
		Frontal orbital cortex	L	3.82	−36	32	−20
		Frontal orbital cortex	L	3.72	−36	28	−22
		Frontal orbital cortex	L	3.60	−30	24	−18
**Carer COAST_1–15_**	503	Precentral gyrus	L	3.90	−32	−4	40
		Superior longitudinal fas	L	3.58	−50	−4	28
		Precentral gyrus	L	3.55	−34	4	32
		Superior longitudinal fas	L	3.54	−42	−8	30
		Postcentral gyrus	L	3.43	−34	−30	44
	485	Brainstem		4.14	2	−34	−26
		Brainstem		4.03	6	−32	−26
		Brainstem		4.02	8	−32	−30
		Brainstem		3.74	6	−46	−36
	428	Inferior frontal gyrus p tri	L	3.81	−38	24	16
		Inferior frontal occipital fas	L	3.76	−34	30	10
		Middle frontal gyrus	L	3.29	−32	30	26

ant: anterior; fas: fasciculus; inf: inferior; L/R: left or right side of the brain; pos: posterior; p tri: pars triangularis; temocc: temporo–occipital, coordinates in Montreal Neurological Institute space.

## Discussion

Despite the importance of functional communication to patients’ activity and participation, the cognitive and neural bases of functional communication, and the best ways to measure it in the clinic and for outcome measures in randomized controlled trials, are not well understood. This study significantly extended our understanding of these issues by assessing the relationships between different functional communication measures, by evaluating the relationship of verbal and non-verbal impairments to functional communication abilities, and by relating patients’ functional communication abilities to their brain lesions.

### How do functional communication measures relate to each other?

Administering two different objective measures of functional communication, the Scenario Test and the ANELT, to a sample of patients covering the whole range of aphasia severity made apparent the relative strengths and weaknesses of these tests. The overall high correlation between the two measures hides the fact that there are considerable floor and ceiling effects in both tests. Being limited to verbal communication, the ANELT can for instance not capture the occasionally remarkable functional communication skills in severely aphasic patients, while patients with relatively intact verbal abilities will obtain an undifferentiated high score on the Scenario Test. Thus, if one wants to grade patients and capture changes in functional communication, for instance within a randomized controlled trial, it is not sufficient to use only one of these measures, unless the sample is restricted in terms of aphasia severity or the scoring of the test is adapted. The German version of the Scenario Test (Nobis-Bosch *et al.*, 2020) for instance contains an extension of the scoring scheme to better account for high (verbal) performers.

The association between the two subjective measures, the patients’ and carers’ version of COAST was still relatively high, whereas the association between the objective and subjective measures was only moderate, in line with previous research (Hilari *et al.*, 2018; Olsson *et al.*, 2019). The lowest correlation emerged between patient’s ratings of their communicative abilities and their performance in the Scenario Test. It seems that some patients tended to underestimate their abilities if they are using non-verbal modes to communicate.

### What are the relationships to underlying cognitive and structural bases?

Given the close but not synonymous relationship between language and communication, it is perhaps not surprising that verbal impairment in general, and phonological abilities in particular, were strongly related to all measures of functional communication. This relationship was most obvious for the ANELT where the regression analyses show that almost all of the variance can be accounted for by direct measures of the patients’ verbal impairment—either in the form of the composite verbal factor score or individual tests of verbal short-term memory such as digit span. Indeed, the variance explained by these regression models is such that they are equivalent to test–retest reliability of the ANELT itself. This would suggest that the time-consuming ANELT assessment could potentially be replaced by much more efficient tests of language ability.

Communication can go beyond language alone, however, and consistent with this fact, we found that non-verbal abilities were critically important, beyond the lesion volume and the verbal measures, when language production was relatively impaired and other modes of communication were allowed, as in the Scenario Test. In these situations, the patients’ non-verbal abilities move to the foreground and retained cognitive skills enable them to use and switch between non-verbal communication strategies. Similarly, a very recent study including only individuals with severe aphasia showed that the relationship between executive abilities (captured by screening tests) and Scenario Test performance was strongest in individuals with hardly any verbal output (Olsson *et al.*, 2019).

The importance of non-verbal cognition became also apparent when patients and carers rated the basic communication abilities. Regression models for this aspect only became significant once overall non-verbal impairment or intermediate non-verbal factor scores, respectively, were included. Beyond this similarity, the bases influencing the carer’s ratings differed in various ways from those of the patient’s ratings and from the tests assessing functional communication. Carers’ ratings of patients’ verbal communication ability for instance, was related to speech quanta (fluency and amount of speech production), which has been previously reported (Fridriksson *et al.*, 2006). Carers’ overall ratings, however, were only significantly related to lesion volume. One interpretation might be that carers’ ratings reflect a more holistic judgement, integrating additional difficulties and resources a patient may have.

The available lesion and impairment-level data were overall considerably less useful for explaining variance in the subjective ratings than in the objective measures. One explanation for this observation is that some items of the rating scale target aspects of communication relying on cognitive abilities that were not tested in detail in this study (for instance arithmetic or praxis). Other items of the rating scale relate to well-being and quality of life, which was not at the centre of this study and thus not further assessed. Interestingly, patient’s basic communication abilities were associated with carers’ well-being. The importance of caregiver burden is increasingly acknowledged, and this finding might be a motivation for further research in this direction.

With regard to the neural bases of functional communication, in the regression analyses we found that lesion load was usually an important variable, suggesting that stroke severity is an important factor. In the brain–behaviour mapping analysis we found that mainly (anterior) temporal as well as inferior frontal regions were related to performance on the more verbally focused ANELT, while the cluster relating to the combined verbal-and-non-verbal Scenario Test also extended into more posterior and dorsal temporo–parieto–occipital regions. Both clusters partially overlap with clusters found for language components (Halai *et al.*, 2017; Schumacher *et al.*, 2019), but the Scenario Test cluster additionally overlapped with the cluster for the non-verbal Shift–Update component (Schumacher *et al.*, 2019), thus mirroring the findings of the regression analyses based on the behavioural data alone. Of note is also that the inferior parietal regions covered by the Scenario Test cluster are associated with apraxia (Goldenberg and Randerath, 2015), which has been shown to play a role in non-verbal communication (Hogrefe *et al.*, 2013). The clusters associated with the subjective measures can also be interpreted as mirroring the findings from the regressions including the behavioural data alone. The cluster along the edge of the core lesion for the Carer COAST reflects its close association with lesion load, while the frontal cluster found to be associated with the Patient COAST might reflect the influence of damage to these regions on executive functioning and self-reported difficulties (Lovstad *et al.*, 2012).

### Where do we go from here?

Our comprehensive approach including objective and subjective measures of functional communication as well as background measures on different levels of specificity enabled us to gain a more thorough understanding of functional communication and its bases. Two further aspects, regarding the sensitivity and demands of functional communication measures, are highlighted by this investigation.

First, the moderate correlation between objective and subjective measures, the floor and ceiling effects in the functional communication tests, and the different patterns of relationships between impairment-level and functional communication measures, underline that there are substantial differences between assessments of functional communication. Moreover, we show that the level of specificity of the independent variables (broad impairment severity, intermediate verbal and non-verbal factor scores, specific tests) as well as of the outcome measures (functional communication (sub)-scores) critically influence which relationships, if any, are found between the measures of interest. To optimally capture potential changes in functional communication following an intervention, it is thus paramount to consider these differences and choose an appropriate type and level (or rather levels) of measurement. Examples of approaches to evaluate more specific aspects of functional communication entail for instance the adaptation of scoring systems (Purdy and Koch, 2006; Nobis-Bosch *et al.*, 2020) or the creation of more specific assessments (Spitzer *et al.*, 2019).

Second, a considerable proportion of the individuals in our sample had sufficient language production abilities to be able to solve the functional communication tests verbally. In everyday life, spoken language is the mode of choice for communication, which is also reflected in the fact that patient’s ratings of communicative ability were heavily based on their language production abilities, and that being able to speak is the most important goal for the majority of patients. Only if the mode of choice is not (sufficiently) available, other abilities, including non-verbal cognition, gain relevance in solving a communicative task. However, it is important to bear in mind that a high score in the Scenario Test or ANELT does not necessarily mean that the individual’s communicative abilities are equal to normal controls or to their pre-morbid abilities. Both tests assess common everyday situations, in which the relevant message can be conveyed with very limited output. The tests may not be very demanding for participants without severe language production deficits (see also Olsson *et al.*, 2019). Moreover, other important aspects of communication, such as pragmatics (Irwin *et al.*, 2002) or discourse (Barker *et al.*, 2017), both more often considered in patients with right-hemispheric brain lesions (Bosco *et al.*, 2017) or traumatic brain injuries (Galski *et al.*, 1998), are not assessed. Thus, further research will be needed to elucidate not only more specific but also more complex aspects of communication (see MacDonald and Johnson, 2005) and their relation to (non-verbal) cognition in stroke aphasia.

To conclude, functional communication is very multifaceted and depends not only on verbal but also on non-verbal abilities. The latter gain importance when a functional communication assessment allows for non-verbal communication (as per its definition) and when verbal abilities are comparably low. Based on our findings, it seems advisable to use more than one measure to assess functional communication, particularly in the context of randomized controlled trials. Moreover, a therapeutic focus on non-verbal cognition might have positive effects on this important aspect of activity and participation. Our thorough approach thus yielded findings that are relevant in two ways: (i) they further elucidated the cognitive and structural bases of functional communication abilities; (ii) they may inform future clinical practice regarding therapy and assessment of functional communication abilities.

## Supplementary Material

fcaa118_Supplementary_Data

## Abbreviations

ANELT =: Amsterdam Nijmegen Everyday Language Test
COAST =: Communication Outcome after Stroke
