# Supplementary material for: The verbal, non-verbal and structural bases of functional communication abilities in aphasia
Source: Brain Commun. 2020 Aug 4;2(2):fcaa118. doi: 10.1093/braincomms/fcaa118 (PMC7660039; doi:10.1093/braincomms/fcaa118)
Supplement: fcaa118_Supplementary_Data [file fcaa118_supplementary_data.pdf]

The verbal, nonverbal and structural bases of functional communication abilities in aphasia

Rahel Schumacher, Stefanie Bruehl, Ajay D. Halai, Matthew A. Lambon Ralph

SUPPLEMENTARY MATERIAL

Contents

Supplementary Table 1 3

Supplementary Table 2 4

Supplementary Table 3 5

Supplementary Figure 1 6

Supplementary Table 1. Participant background information and functional assessment test scores.

Subject	Age	Sex	Edu-cation	years post-stroke	lesion volume	BDAE classification	test interval*	verbal impairment[#]	nonverbal impairment[#]	Scenario Test	ANELT	Patient COAST	Patient COAST 1-15	Carer COAST	Carer COAST 1-15
1	55	m	17	9	11915	Broca	6.4	57.1	37.5	47.0	32.0	57.5	53.3	52.5	45.0
2	55	f	12	12	9767	Anomia	6.2	28.6	12.5	54.0	48.0	70.0	70.0	61.3	53.3
3	71	m	11	8	8788	MNF	6.1	42.9	43.8	53.0	40.0	42.5	41.7	48.8	50.0
4	61	m	11	17	18392	Broca	6.1	50.0	31.3	54.0	37.0	66.3	66.7	n/av	n/av
5	72	m	12	10	41379	Global	5.3	92.9	90.9	17.0	15.0	58.8	53.3	41.3	33.3
6	47	m	11	6	8437	Anomia	3.0	28.6	31.3	53.0	42.0	87.5	85.0	n/av	n/av
7	76	m	11	4	22732	MNF	3.1	78.6	87.5	24.0	14.0	56.3	58.3	48.8	45.0
8	50	f	11	8	6975	Anomia	3.0	64.3	43.8	51.0	36.0	71.3	71.7	n/av	n/av
9	79	f	11	7	13577	Anomia	2.9	64.3	70.0	51.0	35.0	76.3	76.7	65.0	60.0
10	63	f	19	6	9159	Anomia	2.9	42.9	6.3	51.0	42.0	68.4	78.3	n/av	n/av
11	80	m	13	5	34242	MNF	3.0	71.4	43.8	38.0	19.0	78.8	76.7	51.3	51.7
12	71	m	11	4	3311	Anomia	2.9	50.0	18.8	50.0	47.0	63.8	66.7	71.3	66.7
13	62	m	11	5	16433	Anomia	2.9	50.0	18.8	52.0	40.0	61.3	58.3	n/av	n/av
14	70	m	13	7	33239	Global	2.9	100.0	31.3	29.0	11.0	51.3	46.7	48.8	50.0
15	52	m	13	8	22948	Anomia	2.7	78.6	31.3	51.0	25.0	66.3	65.0	53.8	60.0
16	48	f	16	4	3897	Conduction	2.6	71.4	37.5	50.0	29.0	40.0	46.7	50.0	50.0
17	84	m	10	3	12131	Broca	2.6	78.6	44.4	52.0	n/av	n/av	n/av	n/av	n/av
18	46	f	13	5	18948	Anomia	2.2	57.1	18.8	52.0	37.0	61.3	65.0	36.3	40.0
19	75	f	11	6	23863	TMA	2.2	57.1	31.3	52.0	32.0	70.0	68.3	68.8	61.7
20	76	f	11	15	12057	MNF	2.1	85.7	68.8	35.0	18.0	62.5	63.3	53.8	53.3
21	45	f	16	3	175	Anomia	1.8	21.4	25.0	52.0	47.0	67.5	76.7	n/av	n/av
22	66	m	11	4	33239	MNF	1.8	85.7	50.0	47.0	20.0	48.8	45.0	42.5	31.7
23	69	m	11	5	31317	MNF	2.1	92.9	37.5	24.0	10.0	72.5	70.0	60.0	65.0
24	81	m	11	6	33678	MNF	1.8	100.0	57.1	19.0	n/ad	n/ad	n/ad	48.8	46.7
25	47	m	11	3	10409	Anomia	1.8	42.9	31.3	51.0	41.0	65.0	66.7	n/av	n/av
26	59	f	11	24	12699	Anomia	1.7	71.4	37.5	51.0	38.0	58.8	61.7	n/av	n/av
27	68	m	11	2	4879	Conduction	1.6	50.0	18.8	45.0	32.0	61.3	60.0	67.5	61.7
28	53	m	11	7	37822	Global	1.6	100.0	25.0	36.0	10.0	62.5	58.3	32.5	35.0
29	88	m	9	2	8528	Anomia	1.6	57.1	41.7	51.0	37.0	73.8	66.7	58.8	61.7
30	75	m	11	11	36877	Broca	1.6	92.9	73.3	38.0	n/ad	60.0	58.3	46.3	50.0
31	67	m	17	2	6557	Conduction	1.2	64.3	31.3	49.0	36.0	62.5	56.7	53.8	51.7
32	57	m	16	2	6974	Anomia	1.1	57.1	12.5	50.0	39.0	82.5	83.3	73.7	75.0
33	66	m	10	7	6607	Anomia	0.9	50.0	6.3	54.0	42.0	68.8	73.3	78.8	76.7
34	50	m	19	2	4538	Anomia	0.8	35.7	6.3	53.0	42.0	78.8	80.0	83.8	81.7
35	51	m	11	3	14681	Anomia	0.4	35.7	37.5	52.0	46.0	87.5	88.3	81.3	71.7
36	56	f	11	2	10081	MNF	0.4	100.0	50.0	45.0	10.0	31.3	36.7	71.3	73.3
37	69	m	12	7	37907	Broca/ MNF	0.4	85.7	50.0	40.0	18.0	33.8	33.3	48.8	48.3
Mean	63.8		12.4	6.5	17004.3		2.5	64.7	37.6	45.2	31.4	63.6	63.6	57.1	55.4
SD	12.0		2.5	4.6	11994.2		1.6	22.6	21.0	10.5	12.3	13.3	13.5	13.4	13.2

Notes: f = female, m= male, MNF = Mixed Nonfluent, n/av = not available (organisational), n/ad = not administered (based on performance in other tests)
*time (years) between assessment time points 1 and 2, see also Supplementary Table 2; [#] impairment = percentage of impaired test scores (more than 1.5 standard deviations below the mean of controls), based on all available tests, see also Supplementary Table 2

Supplementary Table 2. **Tests, components, and loadings from the principal component analysis including the verbal and nonverbal background tests.**

	**Test measure**	**Phonology**	**Semantics**	**Speech Quanta**	**Shift-Update**	**Inhibit-Generate**	**Speed**
v	Digit span forward[1]	**.898**	.170			-.139	
v	Repetition[1]	**.844**	.181	.138	.246		.116
v	Naming[1]	**.827**	.302	.148	.166		.237
v	Digit span backward[1]	**.775**		.382			
v	Spoken comprehension[1]	**.630**	.459	.168	.441		.103
v	Minimal Pairs[1]	**.615**		-.228		**.572**	
v	Word-picture matching[1]	**.562**	**.611**	.190	.189		-.208
v	Synonym judgment[1]	.420	**.758**	.317	.244		
v	Camel & Cactus Pictures[1]	.334	**.717**		.165	.491	
nv	Design Fluency[2]		**.595**	.177	.222	.431	.279
v	Cookie Theft Token[1]		.214	**.871**	.104	.188	-.108
v	Cookie Theft MLU[1]	.304	.221	**.792**	.328		
	Cookie Theft WPM[1]	.415		**.652**	.155	.108	-.115
nv	Brixton correct[1]	.302		.142	**.782**	.252	-.171
nv	Tower of London planning[2]			.292	**.745**		.369
nv	Divided accuracy[2]	.248	.258		**.708**	.303	
nv	Trail Making numbers[2]	.129	.350	.197	**.683**		.243
nv	Kramer categories[2]		.131	.341		**.778**	
nv	GoNoGo accuracy[2]	-.219			.259	**.712**	.208
nv	Raven B[1]		.140		.334	**.694**	.288
nv	GoNoGo RT[2]	.262				.145	**.842**
nv	Alertness RT[2]	-.112		-.226	.124	.260	**.705**

Notes: v (verbal) and nv (nonverbal) in the leftmost column indicates if the respective test was used to compute the verbal or nonverbal impairment score (see also note in Supplementary Table 1). Superscript numbers indicate in which testing phase the assessment was administered. MLU = mean length of utterance, RT = reaction time, WPM = words per minute, loadings > 0.5 are printed in bold, loadings < 0.1 are not shown

Supplementary Table 3. COAST components and item loadings from principal component analysis, separately for patient and carer versions.

		Patient COAST						Carer COAST				
Nr	Item	verbal communi-cation	improvement & participation	basic communi-cation	confidence & mood	written language & numbers	hobbies	severity	own quality of life	written language & numbers	basic communi-cation	complex interactions
1	yes-no	.121	.279	**.802**			.192			.175	**.760**	.141
2	use other ways	.170		**.703**	.161	.199	-.404	**.707**	.177	.266	.338	-.147
3	chat familiar	**.742**	.206	.105	.149			**.889**	.144	.163		
4	chat unfamiliar	**.602**	.333		.479		.179	**.678**		.484		.235
5	chat group	**.710**	.234		.385	.160		**.660**	.283			.561
6	express long sentences	**.795**		.275		.255	-.122	**.802**		.116	.140	
7	understand simple	**.510**	.185	.238	-.115		**-.580**			**.630**	.542	.312
8	show not understand	.174	.452	**.653**					.186		**.843**	
9	change subject	**.799**		.131		.307	.121	.111			.307	**.803**
10	reading	.172	.119		.173	**.796**		.162		**.841**		
11	writing	.211		.273	-.202	**.671**		.357		**.643**	.221	-.191
12	money				.364	**.730**	.179	-.111	.217	**.794**		.105
13	changed since stroke		**.847**	.119		.355		**.753**	.272	-.129		
14	communication now	.325	**.733**	.171	-.103	.124	-.335	**.830**				.120
15	influence confidence			-.101	**.848**			**.590**	-.132	.470		
16	family life	.326	**.723**	.312	.266	-.106	.104	**.667**	.463	.128	-.127	.111
17	social life	.145	**.669**	-.113	.313		.330	.402	**.782**		.307	-.112
18	interest hobbies	.264	.209	.213	.101	.160	**.832**		**.895**			.114
19	worried unhappy	.166	.128	.303	**.761**	.166			.459	.225	-.213	**.653**
20	quality of life	.115	**.708**	.366	.291	-.122		.164	**.834**		.158	.251

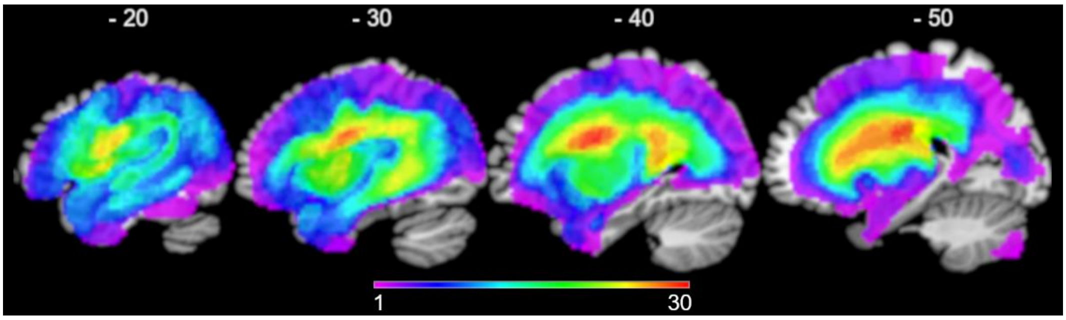

Supplementary Figure 1. Overlap of the 37 patients' lesions.
